# Role of antimicrobial peptide cathelicidin in thrombosis and thromboinflammation

**DOI:** 10.3389/fimmu.2023.1151926

**Published:** 2023-04-05

**Authors:** Qing Zhang, Qurrat Ul Ain, Christian Schulz, Joachim Pircher

**Affiliations:** ^1^ Medizinische Klinik und Poliklinik I, Klinikum der Universität München, Ludwig-Maximilians- Universität, Munich, Germany; ^2^ Partner Site Munich Heart Alliance, DZHK (German Centre for Cardiovascular Research), Munich, Germany

**Keywords:** thrombosis, immunothrombosis, cathelicidin (LL-37), platelets, thromboinflammation, LL-37

## Abstract

Thrombosis is a frequent cause of cardiovascular mortality and hospitalization. Current antithrombotic strategies, however, target both thrombosis and physiological hemostasis and thereby increase bleeding risk. In recent years the pathophysiological understanding of thrombus formation has significantly advanced and inflammation has become a crucial element. Neutrophils as most frequent immune cells in the blood and their released mediators play a key role herein. Neutrophil-derived cathelicidin next to its strong antimicrobial properties has also shown to modulates thrombosis and thus presents a potential therapeutic target. In this article we review direct and indirect (immune- and endothelial cell-mediated) effects of cathelicidin on platelets and the coagulation system. Further we discuss its implications for large vessel thrombosis and consecutive thromboinflammation as well as immunothrombosis in sepsis and COVID-19 and give an outlook for potential therapeutic prospects.

## Introduction

Thrombosis and thrombo-embolism are common causes of death and major health issues worldwide (1). Thrombosis can occur in arteries, veins and the microcirculation, and thus can affect all parts of the cardiovascular system. While arterial thrombosis (commonly associated with atherosclerosis) can result in myocardial infarction, peripheral artery disease and stroke, deep vein thrombosis and pulmonary embolism are major complications of thrombus formation in the venous system. Microvascular thrombosis is triggered mainly in the setting of septic or sterile inflammation, as recently demonstrated impressively in COVID-19 infection, where thrombo-embolic complications turned out as main drivers of mortality (2, 3). For a long time, platelets and the coagulation system have been considered the main players in thrombosis and eventually developed successful targets of therapeutic approaches. However, current antithrombotic strategies (i.e. platelet inhibition and anticoagulation) also affect physiological hemostasis and thereby increase bleeding risk (4). Over the last decade inflammation has been established as a hallmark in the pathophysiology of thrombosis (5). Today, thrombotic and inflammatory processes are considered as inseparably linked and regulated by a complex interaction between immune cells, platelets, and soluble factors (5). While inflammatory diseases constitute a risk factor for both arterial (6–8) and venous thrombosis (9, 10), primary thromboembolic events can directly induce local tissue inflammation and a systemic inflammatory response (11–14). Crosstalk between thrombosis and inflammation in some settings can also be beneficial, as formation of thrombi inside blood vessels can support the immune system facilitating pathogen recognition and destruction, a process termed immunothrombosis (15).

Cathelicidins are antimicrobial peptides that form an effective component of the innate immune system (16–18). Various cells can release these peptides with neutrophils constituting the major source in the blood stream (16, 19–21). Neutrophil-activation is a key feature of thromboinflammation and cathelicidin is abundantly released and able to influence both inflammation and thrombosis (5, 22, 23). This article summarizes recent findings of how cathelicidin affects different aspects of thrombotic processes and highlight its consequences for thrombosis and thromboinflammatory disorders. Eventually, we also discuss these neutrophil-derived antimicrobial peptides as possible therapeutic targets.

## General features of cathelicidin

### Structure and function

Cathelicidins are antimicrobial peptides usually 10–50 residues in length and constitute a crucial component of the innate immune response (16). Though the mature form of the peptides is diverse in length, composition, net charge, and structure, the organization of the coding sequence is well conserved across species even beyond mammals including several vertebrates (24–26). Overall, the biological structure of cathelicidin peptides is diverse and complex, reflecting the many different roles that these peptides play in the immune system. The molecules are generally characterized by a net positive charge and a high proportion of hydrophobic amino acids and share some common structural features (16, 17, 27). Cathelicidins often contain a conserved region called the cathelin domain (28), which is thought to play a role in regulating the activity of the peptide (29). Further, many cathelicidins are alpha-helical peptides, that allows interaction with the membranes of bacterial cells and disrupture of their integrity (30, 31).

Humans express only one cathelicidin, hCAP18 gene is transcribed to a precursor peptide, which is extracellularly cleaved into the active form, that is called LL-37 because it consists of 37 amino acids. The mouse analogue is referred to as cathelicidin-related antimicrobial protein (CRAMP) (16). LL-37 is mainly expressed by immune cells (mainly neutrophils, macrophages, dendritic cells, and natural killer cells), but also by epithelial cells of the skin, eyes, gastrointestinal, genital and respiratory tract (32–34). It is released upon pathogen-mediated (bacteria, viruses, fungi, parasites) endoplasmatic reticulum stress (35), and also by NF-kB-induced inflammatory signals (16, 36), while active vitamin D and several other factors, such as short-chain fatty acids and some cytokines induce cathelicidin transcription (33, 37, 38).

Cathelicidin next to physical interaction with negatively charged membranes can directly or indirectly activate a variety of surface receptors or intracellular targets that are structurally unrelated (16, 39). The most studied receptor interacting with human cathelicidin is formyl peptide receptor like-1 (FPR2), a G-protein-coupled receptor with downstream effects on chemotaxis and angiogenesis (40). Further chemokine (C-X-C motif) receptor 2 (CXCR2), MrgX2, EGFR, IGF1R, or purinergic receptors P2X7 ionotropic receptor and P2Y11 have been associated to cathelicidin (16, 41, 42). After binding to nucleic acids, cathelicidin can enhance cell responses to self-nucleic acids released from damaged and dying cells, by permitting recognition by intracellular recognition systems such as Toll-like receptor (TLR) mitochondrial antiviral-signaling protein (MAVS) and stimulator of interferon genes (STING) (24, 43). Downstream pathways of cathelicidin signaling result in transcription and translation, including the modulation of NF-κB inhibitor-α (IκBα) and several kinase pathways (e.g. mitogen-activated protein kinases (MAPKs) p38, extracellular signal-regulated kinase 1 and 2, JUN N-terminal kinase (JNK) and phosphoinositide 3-kinase) (24, 44–46).

### Antimicrobial and immune-modulatory effects

The first and most extensive investigated function of cathelicidin is its direct antimicrobial activity (44, 47, 48). The cationic and amphophilic character allows direct binding to negatively charged pathogen membranes or nucleic acids (24, 49, 50). These physical properties are prerequisites to induce membrane permeability, pore formation and eventually disruption leading to effective killing of pathogens, which makes cathelicidin a crucial player in the first line of immune defense. Cathelicidin is also effective against viral infection by direct interaction with viral particles and consecutive destabilizing their envelopes (51–54). Recent studies reported a strong interaction with the SARS-CoV2 receptor binding domain (RBD) of the spike protein, and cathelicidin thereby reduced the binding capacity of the cellular SARS-CoV2 receptor ACE2 (55).

Next to their antimicrobial activity, cathelicidin exerts pleiotropic effects on different cell types including both pro- and anti-inflammatory effects (16, 24, 44, 56). These could be explained by their strong affinity to bind other molecules and thereby modulate their function, resulting in enormous variations of the net effects, that can either be beneficial or detrimental in different pathophysiological context and tissue environments. Immune-modulatory effects of cathelicidin most importantly contain enhanced cellular killing capacities e.g. in neutrophils or T-cells (57–60), degranulation of mast cells (61–65), differentiation and polarization of immune cells such as pro-inflammatory macrophage differentiation (66–68), leukocyte recruitment (56, 69), neutralization of bacterial molecules that normally induce proinflammatory immune responses such as LPS (36, 41, 56, 70–73) and induction of type I interferon response (24, 74).

## Thrombo-modulatory mechanisms of cathelicidin

### Direct effects on platelets

Platelets play a central role in thrombus formation in arteries, veins and microvessels. They interact frequently with immune cells to propagate thromboinflammation (5). Platelets are exposed to released cathelicidin locally at sites of thrombus formation but also systemically in the blood stream. Interestingly, data on direct effects of cathelicidin on platelets are scarce.

**Figure 1:**
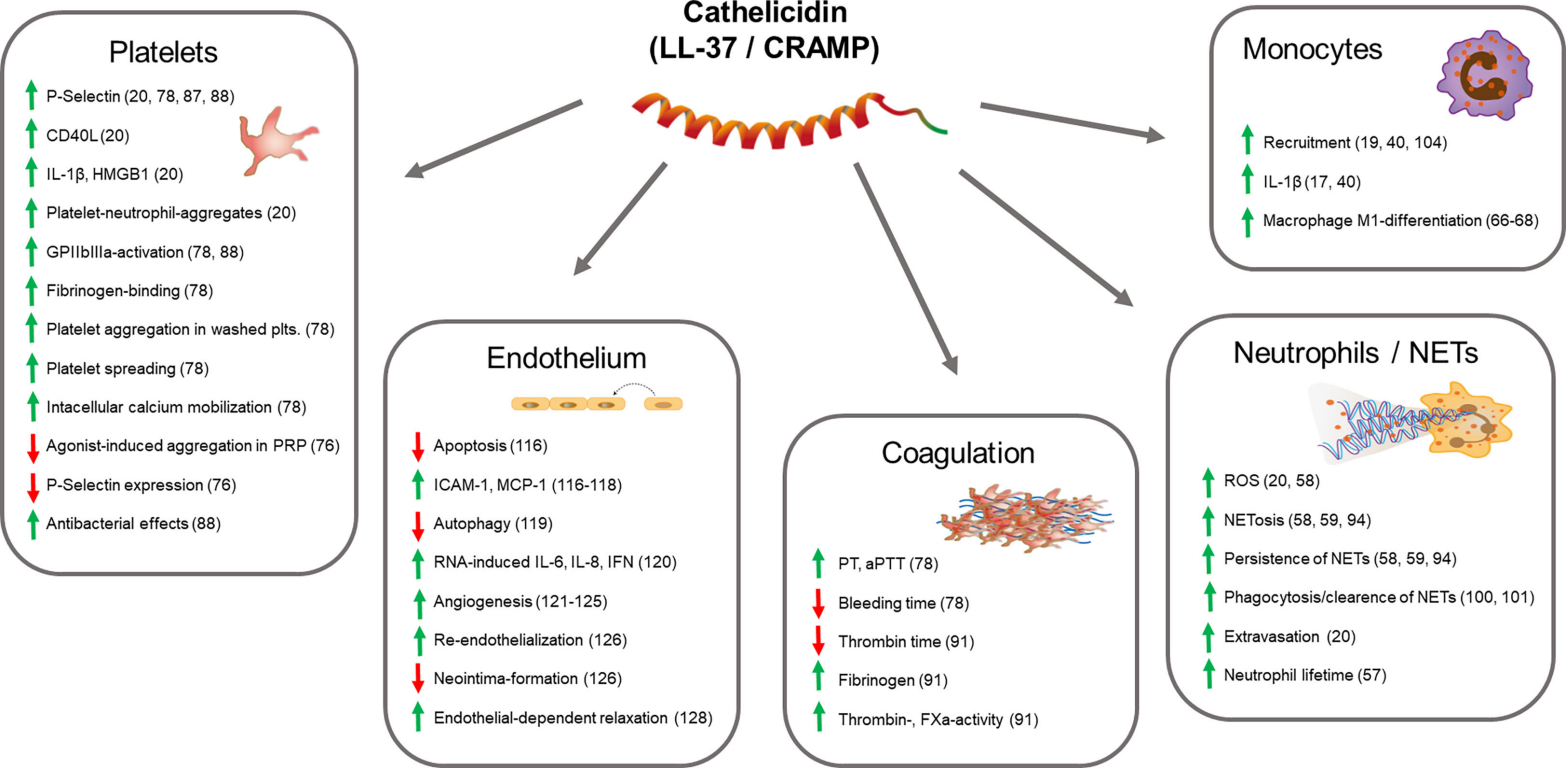
Thrombo-modulatory mechanisms of cathelicidin. The scheme displays effects of cathelicidin on cell types (platelets, endothelial cells, neutrophils, monocytes) and systems (coagulation) that are directly involved in thrombosis and thromboinflammation. Arrows indicate up-/down-regulation or increase/decrease of molecules or functions, respectively. References are indicated in brackets.

In a first study from 2015 investigating possible side effects of cationic antimicrobial peptide-based therapeutic strategies, P-selectin exposure on platelets was not observed after *in vitro* treatment of human platelet rich plasma (PRP) with LL-37 in concentrations from 0.025 mg/mL to 0.1 mg/mL (approximately 5-20 µM) (75). Another study reported inhibitory effects of LL37 on agonist-induced (ADP, U46619, collagen and thrombin at medium dose concentrations) expression of P-Selectin, platelet aggregation and fibrinogen-triggered platelet spreading of isolated human platelets. Mechanistically these observations were linked to reduced phosphorylation of Akt- and Src-kinases, however, cytotoxicity should be taken into consideration since *in vitro* concentrations of LL-37 peptide were in the range of 0.1 mM to 1.2 mM (76). Importantly, cytotoxic effects on eukaryotic cells have been described under culture conditions in the presence of 0.1 mM of LL-37 (77). Two studies published in 2018 more extensively focused on cathelicidin effects exerted on platelets at lower concentrations (20, 78), which are more likely to be achieved locally in inflammatory settings *in vivo* (47, 79–82). Pircher et al. reported that LL-37 in isolated washed human platelets dose-dependently induced alpha degranulation (P-selectin and CD40L surface expression and release) as well as release of IL-1β and HMGB1 (high mobility group box1) starting at 5 µM, which promoted platelet-neutrophil-aggregate formation and neutrophil activation. The suggested molecular mechanism involves glycoprotein VI receptor and downstream signaling through protein tyrosine kinases Src/Syk and phospholipase C. Blockade of other possible receptors for cathelicidin (as described in other cell types) such as FPR1, FPR2, or purinergic P2X7 did not alter LL-37-dependent platelet activation. Interestingly, LL-37 at the same concentrations did not induce GPIIb/IIIa-activation, and did not affect fibrinogen-dependent platelet spreading or platelet aggregation in PRP (both spontaneous and agonist-induced). In line with these findings, Salamah et al. reported comparable effects on LL-37-induced alpha degranulation in isolated platelets (78). Additionally, they observed increased fibrinogen binding, platelet aggregation and spreading. Interestingly, LL-37 hereby induced intracellular calcium mobilization comparable to that induced by cross-linked collagen-related peptide (CRP-XL 1 mg/mL), albeit with faster kinetics. Moreover, this study found low levels of LL-37 stored in platelet granules which were, in analogy to neutrophil responses, released upon activation indicating auto- and paracrine mechanisms of activation. Mechanistically, in mice CRAMP bound to formyl peptide receptor 2 (FPR2/ALX)/Fpr2/3, which is an orthologue to human FPR2/ALX, a Gi-coupled receptor for LL-37/CRAMP on immune cells (83, 84), whose expression has also been reported in megakaryocytes and human and mouse platelets (85, 86). Activation of FPR2/ALX/Fpr2/3 on platelets can increase P-selectin secretion and fibrinogen binding by reducing cAMP-dependent signaling that is known as an inhibitor of platelet functions (78). Accordingly, plasma of mice with psoriasis (that show significantly increased levels of CRAMP) considerably activated wildtype platelets but failed to do so in Fpr2/3-deficient mice (87). In addition to classical platelet activation, both LL-37-stimulated human platelets but also platelet intrinsic cathelicidin showed considerable antibacterial activity *in vitro* characterized by increased binding and killing of bacteria (88). In summary, cathelicidin is a potent activator of platelets that contributes to thromboinflammation (**Figure 1**).

### Direct effects on coagulation and bleeding

The plasmatic coagulation system is an essential factor for thrombus stabilization as well as its resolution. Few studies have investigated possible interactions of cathelicidin and coagulation pathways. In an *in vitro* study that analyzed interactions of cathelicidin and related components with blood cells, LL-37 at concentrations of 50 µg/mL did not influence PT (prothrombin time) and aPPT (activated partial thromboplastin time) in poor platelet plasma, while at higher concentrations (0.2 mg/mL) both PT and aPTT were significantly prolonged (75). However, latter concentrations are unlikely to be reached locally *in vivo* (47, 80, 82). At lower concentrations LL37-dependent alterations in whole blood coagulation were not observed, neither in clotting time nor in clot thickness (75). Mice deficient in hematopoietic CRAMP did not show differences in both extrinsic or intrinsic coagulation ex vivo as assessed by ROTEM thrombelastometry (20). In mouse tail bleeding assays, injection of human LL-37 reduced bleeding time (78), however, no changes were observed in mice deficient for hematopoietic CRAMP (20). In summary, while cathelicidin exerts various effects on platelet functions, the peptides have minimal (if any) effects on blood coagulation at concentrations expected *in vivo* in the absence of inflammatory conditions or infections. New light has been shed on the effects of cathelicidin on the coagulation system in the context of Covid-19 (89), which is associated thromboembolic complications such as pulmonary embolism (2, 3, 90). In this condition levels of LL-37 were negatively correlated with thrombin time and positively related to plasma fibrinogen level suggesting pro-coagulatory effects. Indeed, LL-37 (0-50 ug/mL) increased activity of thrombin and FXa (Factor Xa), fibrinogen, and prothrombin, respectively (91). Further, LL-37 might decrease heparin effects by binding to heparin due to its cationic and amphipathic properties (92), which might influence coagulation. In summary, effects of cathelicidin on the coagulation system are not clear yet with dose-dependency possibly playing a role (**Figure 1**).

### Effects on neutrophil activation and NETs (Neutrophil extracellular traps)

Next to direct effects on platelets or plasmatic coagulation, cathelicidin might modulate thrombosis *via* immune-cell-mediated mechanisms. Neutrophils play a crucial role in venous and arterial thrombosis as well as in immunothrombosis (15, 22, 93). Beside representing the main source of blood cell-derived cathelicidin neutrophil themselves can be activated by human LL-37 in an auto- and paracrine fashion (24). LL-37 potentiates neutrophil respiratory burst and induces NETosis partially through FPR2 (58, 59, 94). Hereby LL-37 translocates towards the nucleus and can disrupt the nuclear membrane (58). Neutrophil extracellular traps (NETs) are released by neutrophils upon activation and a potent mechanism to capture invading pathogens (95). Besides that, NET formation can stimulate coagulation through multifaceted mechanisms including NET-mediated platelet activation, disintegration of tissue factor pathway inhibitor (TFPI), and direct binding of vWF and activating factor XII (15, 96). Cathelicidin is a major component of neutrophil secondary granules and can be released and adhere to NETs (97, 98). In arterial thrombi, extracellular cathelicidin was abundantly associated with NETs (20). While NETs could simply serve as scaffold for cathelicidin to approach pathogens, the specific role of cathelicidin within NETs has been controversially discussed. LL-37 has been reported to be essential for NET survival and persistence by protecting neutrophil DNA from cleavage by bacterial nucleases (58, 59, 94), however, other studies suggested that LL-37 could also help clean NETs by binding to DNA and condensing it to denser assemblies for more effective phagocytosis by macrophages (99, 100). Further, cathelicidin-induced platelet activation led to increased platelet-neutrophil-interaction and thereby enhanced neutrophil ROS production and NETosis. Eventually, CRAMP-activated platelets enhanced injury-induced neutrophil extravasation in mice cremaster muscle venules (20). Thus, cathelicidin associate with extracellular nucleosomes that affect NET functions and provide a platform for interactions with adjacent cells or pathogens (**Figure 1**).

### Effects on monocytes and other immune cells

Monocytes are important cells of the host defense system, but also play a role in thrombotic processes (15). Mechanistically, they express plenty of tissue factor once they are activated, which initiates the coagulation system (101, 102). Cathelicidin not only serves as chemoattractant for neutrophils and monocytes (17, 40) but can upregulate the expression and release inflammatory factors from monocytes, such as IL-1β (17). *In vivo*, cathelicidin recruits classical monocytes to the arteriolar endothelium in the mouse cremaster muscle through formyl-peptide receptor 2 (FPR2), a chemotactic receptor (19, 40, 103). Overall, might contribute to immunothrombosis and thromboinflammation in a monocyte-dependent manner.

In addition to effects on myeloid cells, cathelicidin can potentiate T-helper cell differentiation into a Th17 phenotype (104), thereby linking innate and adaptive immune responses. Th17/IL-17A-mediated inflammatory response in turn has been associated to a pro-atherosclerotic phenotype (105) and prothrombotic effects in autoimmune diseases (106). Further, stimulation of PBMCs (peripheral blood mononuclear cells) with LL-37 was associated with reduced programmed cell death protein 1 (PDCD1) mRNA expression in patients with acute coronary syndrome (107) (**Figure 1**).

### Effects on endothelial cells

Next to blood and immune cells the vascular endothelium is an essential regulator of hemostasis and functional endothelial cells continuously release antithrombotic mediators such as nitric oxide and prostaglandin to prevent blood clotting (108–111). While disruption of the endothelium leads to exposure of subendothelial collagen to the blood stream with consecutive rapid thrombus formation, also endothelial dysfunction is associated with a prothrombotic state and additionally leads to progressive vascular inflammation (22, 112–114). Activated endothelial cells express adhesion molecules including P-selectin, E-selectin, vWF, ICAM, and VCAM that recruit neutrophils, platelets, and monocytes (114). Effects of cathelicidin on endothelial cells are not understood in detail and are probably context-related. While LL-37 was shown to inhibit apoptosis of endothelial cells by neutralizing LPS (115), endothelial cells showed LL-37-dependent NF-kappaB-activation, expression of ICAM-1 and monocyte chemoattractant protein 1 (115–117). Further, LL-37 induces autophagy in endothelial cells but enhances cell death in autophagy-dysfunctional conditions, that plays a role in the pathogenesis of atherosclerosis (118). *In vitro*, LL-37 rapidly activated endothelial IL-6 gene expression, but this effect was temporary and not observed after several hours. However, LL-37 strongly boosted upregulation of IL-6, IL-8 and interferon beta expression induced by double-stranded RNA-analogon polyinosinic:polycytidylic acid (polyI:C). In contrast, while endothelial uptake of viral DNA was facilitated by LL-37, DNA-induced inflammatory response was abrogated (119). While abovementioned rather pro-inflammatory effects are clearly related to prothrombotic consequences, other studies describe cathelicidin-dependent effects that would rather decrease thromboinflammation. So, LL-37 has been shown to induce endothelium-dependent angiogenesis *via* FPR2-receptor and to stimulate proliferation and formation of vessel-like structures in cultivated endothelial cells (120). Similarly, the murine homologue CRAMP induced prostaglandin-dependent angiogenesis *in vivo* (121). Immobilized forms of cathelicidin *in vitro* showed mitogenic effects on endothelial cells that were comparable to those of vascular endothelial growth factor (VEGF) (122). Further, LL-37 induced tumor stromal cells to express strong pro-angiogenic factors that eventually facilitated tumor progression (123). Consequently therapeutic approaches of topical application of recombinant LL37 promoted wound healing through vascularization (124). In line with this, neutrophil-born LL-37/CRAMP promoted re-endothelization potentially through the same receptor (125). This limited neointima formation at sites of endothelial injury, mediated by recruitment and increased survival of endothelial outgrowth cells (125, 126). Further, LL-37 promoted endothelium-dependent relaxation in human omental veins mediated by FPR2-dependent release of nitric oxide and endothelium-derived hyperpolarizing factor (EDHF) but not prostanoids (127) (**Figure 1**).

## Cathelicidin in thrombotic and thromboinflammatory diseases

### Release in thrombosis and thromboinflammation

Several cell types involved in thromboinflammation such as neutrophils, endothelial cells and macrophages express cathelicidin (16), with neutrophil-born likely to be the main source particularly in acute thrombotic processes (20). Neutrophil-activation is found in both pathogen-triggered immunothrombosis as well as sterile thrombosis with consecutive thromboinflammation. In microbial infection as well as sterile inflammation pathogen- or damage-associated molecular patterns (PAMPs or DAMPs, respectively) as well as many inflammatory cytokines (e.g. IL-8, IL-6, IFN-γ, TNF α) and chemokines activate neutrophils and trigger cathelicidin release (24, 31, 128–133). In thrombosis activated platelets interact with neutrophils *via* P-selectin and P-selectin glycoprotein ligand 1 as well as glycoprotein Ib and macrophage-1 antigen, which mediate reciprocal activation directly *via* physical cell interactions as well as release of soluble mediators (23, 102, 134–136). Inflammatory signals lead to cathelicidin gene expression in response to endoplasmic reticulum (ER) stress and NF-κB activation (35). Additionally, cathelicidin stored as inactive precursor in azurophilic granules can be rapidly released by degranulation during immune responses (132, 137). Cathelicidin can be released, but also be exposed and bound to surface membranes and extracellular nucleosomes such as NETs as described above, which is a key feature in thrombosis (20, 97, 100, 138).

Although the effects of cathelicidin on different cell types have been extensively studied *in vitro*, the dynamics of release and concentration *in vivo* remain speculative. This is in part due to limitations in rapid local sample acquisition as well as technical *in vitro* handling, since the positively charged peptides readily stick to negatively charged surfaces such as cell membranes and also laboratory tubes. The concentrations of cathelicidin shown to have effects on platelets and other blood cells *in vitro* are not reached by those measured in the systemic circulation (79), although higher local concentrations have been found in bronchoalveolar lavage (BAL) fluid and at different mucosal sites (47, 80, 82). Although cathelicidin is considered to have a short half-life, it is very likely that relevant amounts of neutrophil-derived cathelicidin are exposed to blood components and other cells at sites of thrombosis. Though, neutrophils are considered the main source of cathelicidin in thromboinflammation that have capacity of immediate release of high amounts with influence on acute processes (20), release by other cells such as monocytes/macrophages, endothelial, smooth muscle cells or even platelets (16, 78, 88, 139) might contribute to ongoing inflammation with effects on development of chronic vascular diseases such as atherosclerosis (19, 116) (**Table 1**).

**Table 1:** Cathelicidin expression and proposed function in thromboinflammatory diseases.

Disease	Main findings and mechanism	Ref.
Atherosclerosis	LL-37 detected in human atherosclerotic plaques	(116, 139)
	Decreased lesion size in CRAMP-/- ApoE-/- mice due to reduced recruitment of classical inflammatory monocytes and neutrophils	(19)
	CRAMP-mtDNA complexes aggravate atherosclerosis in ApoE-/- mice	(140)
	T cells reactive to mouse cathelicidin modulate plaque calcification in ApoE-/- mice	(107)
Thrombosis and haemostasis	Neutrophil-derived cathelicidin found in human coronary artery thrombi and FeCl3 induced mouse carotid thrombi	(20)
	Deficiency of (hematopoietic) CRAMP reduced FeCl3-induced carotid artery thrombosis and ligation-induced platelet adhesion	(20, 91)
	IV injection of LL-37 shortened bleeding time in mice	(78)
	IV injection of LL-37 or CRAMP accelerated arterial thrombosis in mice	(91)
	Injection of LL-37 decreased thrombus weight in arterio-venous shunt thrombosis in rats	(76)
Myocardial infarction and ischemia	LL-37-reactive CD8+ effector T-cells are associated with acute coronary events in human	(107)
	Systemic plasma levels of LL-37 transiently decreased in patients with STEMI, but are higher levels in culprit lesion	(141)
	CRAMP protects against cardiomyocyte apoptosis and cardiac I/R injury via activation of Akt and ERK and nuclear export of FoxO3a	(142)
	CRAMP aggravates ischemia-reperfusion injury via TLR4 and NLRP3-inflammasome activation	(143)
	Basal plasma levels of LL-37 associated with lower risk of ischemic events within the first 3 years after STEMI	(144)
	Cathelicidin recruits and retain bone marrow-derived stem/progenitor cells after myocardial infarction	(145–147)
	Cathelicidin-biofunctionalized stent reduced the incidence of in-stent-restenosis due to reduced neointima formation	(125)
	LL-37/CRAMP increases angiogenesis in rabbit hindlimb ischemia and wound revascularizatoin in mice	(120)
Immunothrombosis and sterile thromboinflammatory microvascular diseases	Protective role of cathelicidin in mouse models of sepsis by neutralization of LPS and induction of NETosis	(31, 58, 72, 94, 148, 149)
	Hematopoietic CRAMP-deficient mice protected from acid-induced lung injury and reduced pulmonary NETosis	(20)
	IV injection of high doses of human LL-37 or murine CRAMP induces pulmonary microvascular thrombosis in mice	(91)
	LL-37 strongly induces NETs in systemic lupus erythematosus	(150)
	Strong cathelicidin-dependent platelet activation in psoriasis	(87)
Covid-19	Neutrophil-derived LL-37 inversely correlated with disease severity in Covid19-infection	(151)
	Epithelial LL-37 was upregulated by SarsCov-2 spike protein and elevated in plasma of Covid19-patients	(91)
	Intranasal administration of LL-37 decreased lung infection in mice infected with human ACE2 expressing adenovirus	(55)
	LL-37 levels negatively correlated with thrombin time but positively correlated with fibrinogen level	(91)

### Cathelicidin in atherothrombotic and large vessel disease

Cardiovascular diseases remain the leading cause of mortality worldwide despite current therapeutic interventions. The majority of cardiovascular deaths are caused by myocardial infarction and stroke following rupture of an atherosclerotic plaque and subsequent thrombotic arterial occlusion. Atherosclerosis is nowadays considered an inflammatory disease and immune cells, including neutrophils, play a role in all steps of atherothrombosis (152–156).

In this context, LL-37 has been detected in human atherosclerotic plaques, where it interacts with macrophages and endothelial cells (116, 139). A functional role in the progression of atherosclerosis was identified in ApoE-deficient mice (a commonly used mouse model for atherosclerosis), where cathelicidin deficiency significantly decreased atherosclerotic lesion size (19). Mechanistically neutrophil-derived cathelicidin enhanced recruitment of classical inflammatory monocytes and neutrophils (19). Another study indicated that CRAMP-mtDNA complexes aggravate atherosclerotic lesion formation in ApoE-deficient mice and suggests that LL-37-mtDNA complex acts as a key mediator of atherosclerosis formation (140). Furthermore, T cells reactive to mouse cathelicidin may be involved in modulating plaque calcification in ApoE-deficient mice (107).

Rupture of atherosclerotic plaques is the primary cause for arterial thrombosis and associated mortality in myocardial infarction and stroke. LL-37 was abundant in coronary artery thrombi of patients with acute myocardial infarction as well as mouse arterial thrombi induced by ferric chloride injury (20). In this context cathelicidin derived almost exclusively from neutrophils and was abundantly associated to neutrophil-derived nucleosomes (NETs). Deficiency in hematopoietic CRAMP delayed ferric chloride-induced carotid artery occlusion and decreased thrombus size and stability; similarly, carotid artery ligation-induced platelet adhesion was reduced (20, 91), while bleeding time was unaffected (20). An independent study showed that intravenous injection of LL-37 (20 µM) into mice shortened bleeding time (78). Accordingly, addition of LL-37 (10-50 µM) to human whole blood concentration-dependently increased thrombus formation ex vivo on collagen-coated slides under arterial shear rates (78). Recently, similar effects with accelerated arterial thrombosis in mice were described upon injection of both human LL-37 or murine CRAMP (91). Interestingly in a slightly higher dose (10-15 mg/kg corresponding approximately 50 µM in the blood) LL-37 decreased thrombus weight in a model of arterio-venous shunt thrombosis in rats (76). Future studies should address the role of cathelicidin in venous thrombosis and thromboembolism.

### Cathelicidin in myocardial infarction and ischemia

Though one could assume that cathelicidin could promote acute myocardial infarction, the best example for atherothrombosis, its role in this setting remains unclear. A recent study suggested that the persistence of LL-37-reactive CD8+ effector T-cells may be involved in acute coronary events (107). Observations from clinical studies showed that systemic plasma levels of LL-37 transiently decreased in patients with ST-segment myocardial infarction (STEMI) as compared to patients without or stable coronary artery disease, but were restored within 24 hours (141). However, local plasma levels in the culprit lesion were higher than in the systemic circulation (141). Explanations for these observations are speculative, as excessive trapping of the positively charged peptides at injury sites but also binding to heparin (which is routinely administered early even after suspicion of acute myocardial infarction) are possible. As cathelicidin release is likely to promote acute events, its role for consecutive myocardial injury is controversially discussed, as discrepant impact on ischemia-reperfusion injury have been described, including both protective effects on cardiomyocytes as well as harmful response *via* activation of Akt/ERK and nuclear export of FoxO3a or *via* TLR4- and NLRP3-inflammasome activation, respectively (142, 143). Interestingly, higher basal plasma levels of LL-37 were associated with lower risk of ischemic cardiovascular events within the first 3 years after STEMI (144).

Further, cathelicidin may recruit and retain bone marrow-derived stem/progenitor cells (BMSPC) after myocardial infarction, which potentially could help post-AMI remodeling and recovery (145–147). On the other hand neutrophils after myocardial infarction induce excessive release of platelets and thereby boost the risk of recurrent ischemia (157). Another study showed that LL37/CRAMP increases angiogenesis in rabbit hindlimb ischemia model as well neovascularization of wounds in mice (120). Noteworthy, as neutrophil-born cathelicidin promoted angiogenesis and re-endothelialization, in an experimental model of stent-thrombosis (stenting of occluded arteries is the first-line intervention in acute coronary syndrome) a cathelicidin-biofunctionalized stent reduced the incidence of in-stent-restenosis (125).

### Cathelicidin in immunothrombosis

Immunothrombosis is a critical process in which pathogen-induced inflammation uses microvascular thrombosis to combat infections and defend pathogens (5, 15, 96). Immunothrombosis can facilitate pathogen recognition, compartmentalization, trapping and killing, but also prevent spreading. Mechanistically the process is based on complex and reciprocal interplay between platelets, the coagulation system and innate immune cells. Herein the role of neutrophils is to highlight and their property to undergo NETosis is a key feature to induce thrombosis (96, 158–161). Considering cathelicidin an essential component of neutrophil granules as well as NETs, it might play a central role in this context. Surprisingly its precise role has not been investigated in a specific model of immunothrombosis so far. However, several studies showed that cathelicidin has a protective role in mouse models of sepsis which is mediated by several mechanisms (31, 72, 148). Hereby, next to direct antibacterial properties and neutralization of LPS, LL-37 strongly induced of NETosis in sepsis (58, 94, 149, 162), which in term is a main driver of immunothrombosis. While the term “immunothrombosis” has developed with respect to thrombosis in response to pathogens, similar mechanisms are observed in response to sterile inflammatory conditions such as ARDS (acute respiratory distress syndrome) or autoimmune diseases (5, 163, 164). In a mouse model of sterile lung injury deficiency of hematopoietic cathelicidin reduced pulmonary NETosis and systemic markers of thromboinflammation (20). Interestingly, a recent study described spontaneous microvascular thrombus formation in the lung of mice 10 minutes after intravenous injection of high doses (30 mg/kg) of human LL37 or murine CRAMP (91). However, it remains unclear whether local thrombus formation or thromboembolism was responsible for these observations. Further, LL-37 has shown to be a strong inducer of NETs in systemic lupus erythematosus (150) that correlates with both micro- and macrovascular thrombosis (98, 129, 165). In psoriasis, a systemic autoimmune disease mainly affecting skin and joints, cathelicidin levels are locally but also systemically elevated (60, 87). Strong cathelicidin-dependent platelet activation has been described (87) and may contribute to the prothrombotic state in these patients (166).

### Cathelicidin in Covid-19

The importance of the crosstalk between inflammation and thrombosis got notable attention in the context of the Covid-19-pandemic. Soon after the beginning of the global spread of SARS-CoV-2-virus it became evident that next to acute respiratory distress syndrome, cardiovascular events such as venous thromboembolism, MI and stroke were major causes of fatality in infected patients (2, 3, 90). Numerous studies have highlighted the role of NETosis and thromboinflammation in this context (5, 167–169). Given the outstanding position of cathelicidin at the crossroads of infection, inflammation and thrombosis several studies have focused on a pathophysiological role and speculated with therapeutic prospects (170, 171). Human cathelicidin LL-37 exerted antiviral properties by reducing SARS-CoV-2 binding capacity to its cellular receptor ACE2 (55) and neutrophil-derived LL-37 was inversely correlated with disease severity supporting protective effects in Covid19-infection (151). A study particularly focusing on possible effects of cathelicidin on thrombotic processes, observed that epithelial LL-37 was upregulated by the spike protein and consequently elevated in plasma of Covid-19 patients. LL-37 levels negatively correlated with thrombin time but positively correlated with fibrinogen level. Cathelicidin enhanced platelet activation and activity of coagulation factors Xa (FXa) and thrombin, suggesting that it significantly contributes to prothrombotic state in Covid-19-infection (91).

### Cathelicidin as possible therapeutic target

Ever since cathelicidin has been discovered more than 20 years ago, the promise to take therapeutic advantage of the peptide has shined on the horizon. Particularly its antimicrobial effects have been considered as useful synergism to conventional antibiotics in bacterial infections (31, 48, 172–174). Nevertheless, most of the pre-clinical studies have failed to advance the therapeutic implications for the clinic. One reason for this might be a relatively narrow window of bioavailability, because peptides are quickly broken down by proteases, which limits routes of administration. This problem has not been reliably managed so far even with modern technologies, such as nanoparticle-carriers, which modulate its functions (175, 176). Local applications such as the cornea of the eye or skin lesions may be accessible for therapeutic approaches of cathelicidin-modifying therapies (124, 177, 178). However, systemic interventions are hampered by context-related pleiotropy and complex immunomodulatory effects, and by consecutive unwanted side effects. For example in immunothrombotic settings it is challenging to find a therapeutic range in which cathelicidin successfully combats microbes and on the other hand does not drive excessive thromboinflammation (5, 31). With respect to sterile thrombotic disorders, where antimicrobial properties might be dispensable, a straightforward approach for pharmacologic inhibition of cathelicidin remains still challenging as beneficial effects on ischemic tissues might escape (142, 143, 179). Therefore, to take therapeutic advantage of cathelicidin particularly in thrombosis and thromboinflammation, it is essential to know its precise pathophysiological spatial and temporal role in several tissues. Though, this still will not automatically overcome above-listed problems and make cathelicidin a grateful target in thrombosis, however it could propose interesting new or more easily accessible downstream targets, such as platelet GPVI- and FPR2-receptors or even the P-selectin-PSGL1-axis as master regulator of platelet-neutrophil-interaction (20, 23, 78, 180).

## Conclusion

Cathelicidin – in particular of neutrophil origin – plays an important role in both inflammation and thrombosis, and thereby in principle represents an excellent therapeutic target candidate for thrombosis and thromboinflammation. So far clinical application has been limited by in part poorly understood and highly variable effects on tissues. Further research extending our knowledge on the precise function of cathelicidin in health and disease might overcome limitations and bring advantages for the treatment of inflammatory diseases.

## Author contributions

QZ, QU, and JP did literature research and wrote the review. CS and JP supervised and revised the review. All authors contributed to the article and approved the submitted version.

## References

[1] Tsao CW Aday AW Almarzooq ZI Alonso A Beaton AZ Bittencourt MS. Heart disease and stroke statistics-2022 update: A report from the American heart association. Circulation (2022) 145(8):e153–639. doi: 10.1161/CIR.0000000000001052 35078371

[2] Klok FA Kruip M van der Meer NJM Arbous MS Gommers D Kant KM. Incidence of thrombotic complications in critically ill ICU patients with COVID-19. Thromb Res (2020) 191:145–7. doi: 10.1016/j.thromres.2020.04.013 PMC7146714 32291094

[3] Klok FA Kruip M van der Meer NJM Arbous MS Gommers D Kant KM. Confirmation of the high cumulative incidence of thrombotic complications in critically ill ICU patients with COVID-19: An updated analysis. Thromb Res (2020) 191:148–50. doi: 10.1016/j.thromres.2020.04.041 PMC7192101 32381264

[4] Steg PG Huber K Andreotti F Arnesen H Atar D Badimon L. Bleeding in acute coronary syndromes and percutaneous coronary interventions: Position paper by the working group on thrombosis of the European society of cardiology. Eur Heart J (2011) 32(15):1854–64. doi: 10.1093/eurheartj/ehr204 21715717

[5] Stark K Massberg S. Interplay between inflammation and thrombosis in cardiovascular pathology. Nat Rev Cardiol (2021) 18(9):666–82. doi: 10.1038/s41569-021-00552-1 PMC8100938 33958774

[6] Maradit-Kremers H Nicola PJ Crowson CS Ballman KV Gabriel SE. Cardiovascular death in rheumatoid arthritis: A population-based study. Arthritis rheumatism (2005) 52(3):722–32. doi: 10.1002/art.20878 15751097

[7] Ross R. Atherosclerosis–an inflammatory disease. N Engl J Med (1999) 340(2):115–26. doi: 10.1056/NEJM199901143400207 9887164

[8] Smeeth L Thomas SL Hall AJ Hubbard R Farrington P Vallance P. Risk of myocardial infarction and stroke after acute infection or vaccination. N Engl J Med (2004) 351(25):2611–8. doi: 10.1056/NEJMoa041747 15602021

[9] Mackman N. New insights into the mechanisms of venous thrombosis. J Clin Invest (2012) 122(7):2331–6. doi: 10.1172/JCI60229 PMC3386811 22751108

[10] Smeeth L Cook C Thomas S Hall AJ Hubbard R Vallance P. Risk of deep vein thrombosis and pulmonary embolism after acute infection in a community setting. Lancet (2006) 367(9516):1075–9. doi: 10.1016/S0140-6736(06)68474-2 16581406

[11] King KR Aguirre AD Ye YX Sun Y Roh JD Ng RP Jr.. IRF3 and type I interferons fuel a fatal response to myocardial infarction. Nat Med (2017) 23(12):1481–7. doi: 10.1038/nm.4428 PMC6477926 29106401

[12] Stoll G Nieswandt B. thromboinflammation in acute ischaemic stroke - implications for treatment. Nat Rev Neurol (2019) 15(8):473–81. doi: 10.1038/s41582-019-0221-1 31263257

[13] Rabinovich A Cohen JM Cushman M Wells PS Rodger MA Kovacs MJ. Inflammation markers and their trajectories after deep vein thrombosis in relation to risk of post-thrombotic syndrome. J Thromb Haemost (2015) 13(3):398–408. doi: 10.1111/jth.12814 25495610

[14] Kaptein FHJ Kroft LJM Hammerschlag G Ninaber MK Bauer MP Huisman MV. Pulmonary infarction in acute pulmonary embolism. Thromb Res (2021) 202:162–9. doi: 10.1016/j.thromres.2021.03.022 33862471

[15] Engelmann B Massberg S. Thrombosis as an intravascular effector of innate immunity. Nat Rev Immunol (2013) 13(1):34–45. doi: 10.1038/nri3345 23222502

[16] Vandamme D Landuyt B Luyten W Schoofs L. A comprehensive summary of LL-37, the factotum human cathelicidin peptide. Cell Immunol (2012) 280(1):22–35. doi: 10.1016/j.cellimm.2012.11.009 23246832

[17] Bandurska K Berdowska A Barczynska-Felusiak R Krupa P. Unique features of human cathelicidin LL-37. BioFactors (Oxford England) (2015) 41(5):289–300. doi: 10.1002/biof.1225 26434733

[18] Agerberth B Charo J Werr J Olsson B Idali F Lindbom L. The human antimicrobial and chemotactic peptides LL-37 and alpha-defensins are expressed by specific lymphocyte and monocyte populations. Blood (2000) 96(9):3086–93. doi: 10.1182/blood.V96.9.3086 11049988

[19] Doring Y Drechsler M Wantha S Kemmerich K Lievens D Vijayan S. Lack of neutrophil-derived CRAMP reduces atherosclerosis in mice. Circ Res (2012) 110(8):1052–6. doi: 10.1161/CIRCRESAHA.112.265868 22394519

[20] Pircher J Czermak T Ehrlich A Eberle C Gaitzsch E Margraf A. Cathelicidins prime platelets to mediate arterial thrombosis and tissue inflammation. Nat Commun (2018) 9(1):1523. doi: 10.1038/s41467-018-03925-2 29670076 PMC5906636

[21] Yang D de la Rosa G Tewary P Oppenheim JJ. Alarmins link neutrophils and dendritic cells. Trends Immunol (2009) 30(11):531–7. doi: 10.1016/j.it.2009.07.004 PMC2767430 19699678

[22] von Bruhl ML Stark K Steinhart A Chandraratne S Konrad I Lorenz M. Monocytes, neutrophils, and platelets cooperate to initiate and propagate venous thrombosis in mice in vivo. J Exp Med (2012) 209(4):819–35. doi: 10.1084/jem.20112322 PMC3328366 22451716

[23] Pircher J Engelmann B Massberg S Schulz C. Platelet-neutrophil crosstalk in atherothrombosis. Thromb Haemost (2019) 119(8):1274–82. doi: 10.1055/s-0039-1692983 31254975

[24] Alford MA Baquir B Santana FL Haney EF Hancock REW. Cathelicidin host defense peptides and inflammatory signaling: Striking a balance. Front Microbiol (2020) 11:1902. doi: 10.3389/fmicb.2020.01902 32982998 PMC7481365

[25] Wang G. Structures of human host defense cathelicidin LL-37 and its smallest antimicrobial peptide KR-12 in lipid micelles. J Biol Chem (2008) 283(47):32637–43. doi: 10.1074/jbc.M805533200 18818205

[26] Gogoladze G Grigolava M Vishnepolsky B Chubinidze M Duroux P Lefranc MP. DBAASP: Database of antimicrobial activity and structure of peptides. FEMS Microbiol Lett (2014) 357(1):63–8. doi: 10.1111/1574-6968.12489 24888447

[27] Fjell CD Hiss JA Hancock RE Schneider G. Designing antimicrobial peptides: form follows function. Nat Rev Drug Discov (2011) 11(1):37–51. doi: 10.1038/nrd3591 22173434

[28] Zhu S. Did cathelicidins, a family of multifunctional host-defense peptides, arise from a cysteine protease inhibitor? Trends Microbiol (2008) 16(8):353–60. doi: 10.1016/j.tim.2008.05.007 18632274

[29] Tjabringa GS Aarbiou J Ninaber DK Drijfhout JW Sørensen OE Borregaard N. The antimicrobial peptide LL-37 activates innate immunity at the airway epithelial surface by transactivation of the epidermal growth factor receptor. J Immunol (2003) 171(12):6690–6. doi: 10.4049/jimmunol.171.12.6690 14662872

[30] Zhang LJ Gallo RL. Antimicrobial peptides. Curr biology: CB (2016) 26(1):R14–9. doi: 10.1016/j.cub.2015.11.017 26766224

[31] Nagaoka I Tamura H Reich J. Therapeutic potential of cathelicidin peptide LL-37, an antimicrobial agent, in a murine sepsis model. Int J Mol Sci (2020) 21(17). doi: 10.3390/ijms21175973 PMC7503894 32825174

[32] Schrumpf JA van Sterkenburg MA Verhoosel RM Zuyderduyn S Hiemstra PS. Interleukin 13 exposure enhances vitamin d-mediated expression of the human cathelicidin antimicrobial peptide 18/LL-37 in bronchial epithelial cells. Infect Immun (2012) 80(12):4485–94. doi: 10.1128/IAI.06224-11 PMC3497402 23045480

[33] Gombart AF Borregaard N Koeffler HP. Human cathelicidin antimicrobial peptide (CAMP) gene is a direct target of the vitamin d receptor and is strongly up-regulated in myeloid cells by 1,25-dihydroxyvitamin D3. FASEB J (2005) 19(9):1067–77. doi: 10.1096/fj.04-3284com 15985530

[34] Fagerberg L Hallström BM Oksvold P Kampf C Djureinovic D Odeberg J. Analysis of the human tissue-specific expression by genome-wide integration of transcriptomics and antibody-based proteomics. Mol Cell Proteomics (2014) 13(2):397–406. doi: 10.1074/mcp.M113.035600 24309898 PMC3916642

[35] Park K Elias PM Oda Y Mackenzie D Mauro T Holleran WM. Regulation of cathelicidin antimicrobial peptide expression by an endoplasmic reticulum (ER) stress signaling, vitamin d receptor-independent pathway. J Biol Chem (2011) 286(39):34121–30. doi: 10.1074/jbc.M111.250431 PMC3190812 21832078

[36] Li G Domenico J Jia Y Lucas JJ Gelfand EW. NF-kappaB-dependent induction of cathelicidin-related antimicrobial peptide in murine mast cells by lipopolysaccharide. Int Arch Allergy Immunol (2009) 150(2):122–32. doi: 10.1159/000218115 PMC2814151 19439978

[37] Chen J Vitetta L. The role of butyrate in attenuating pathobiont-induced hyperinflammation. Immune Netw (2020) 20(2):e15. doi: 10.4110/in.2020.20.e15 32395367 PMC7192831

[38] Wang TT Nestel FP Bourdeau V Nagai Y Wang Q Liao J. Cutting edge: 1,25-dihydroxyvitamin D3 is a direct inducer of antimicrobial peptide gene expression. J Immunol (2004) 173(5):2909–12. doi: 10.4049/jimmunol.173.5.2909 15322146

[39] Verjans ET Zels S Luyten W Landuyt B Schoofs L. Molecular mechanisms of LL-37-induced receptor activation: An overview. Peptides (2016) 85:16–26. doi: 10.1016/j.peptides.2016.09.002 27609777

[40] De Y Chen Q Schmidt AP Anderson GM Wang JM Wooters J. LL-37, the neutrophil granule- and epithelial cell-derived cathelicidin, utilizes formyl peptide receptor-like 1 (FPRL1) as a receptor to chemoattract human peripheral blood neutrophils, monocytes, and T cells. J Exp Med (2000) 192(7):1069–74. doi: 10.1084/jem.192.7.1069 PMC2193321 11015447

[41] Yu X Quan J Long W Chen H Wang R Guo J. LL-37 inhibits LPS-induced inflammation and stimulates the osteogenic differentiation of BMSCs *via* P2X7 receptor and MAPK signaling pathway. Exp Cell Res (2018) 372(2):178–87. doi: 10.1016/j.yexcr.2018.09.024 30287143

[42] Zhang Z Cherryholmes G Chang F Rose DM Schraufstatter I Shively JE. Evidence that cathelicidin peptide LL-37 may act as a functional ligand for CXCR2 on human neutrophils. Eur J Immunol (2009) 39(11):3181–94. doi: 10.1002/eji.200939496 PMC3076219 19750480

[43] Takahashi T Kulkarni NN Lee EY Zhang LJ Wong GCL Gallo RL. Cathelicidin promotes inflammation by enabling binding of self-RNA to cell surface scavenger receptors. Sci Rep (2018) 8(1):4032. doi: 10.1038/s41598-018-22409-3 29507358 PMC5838106

[44] Bowdish DM Davidson DJ Hancock RE. Immunomodulatory properties of defensins and cathelicidins. Curr Top Microbiol Immunol (2006) 306:27–66. doi: 10.1007/3-540-29916-5_2 16909917 PMC7121507

[45] Scott MG Davidson DJ Gold MR Bowdish D Hancock RE. The human antimicrobial peptide LL-37 is a multifunctional modulator of innate immune responses. J Immunol (2002) 169(7):3883–91. doi: 10.4049/jimmunol.169.7.3883 12244186

[46] Mookherjee N Wilson HL Doria S Popowych Y Falsafi R Yu JJ. Bovine and human cathelicidin cationic host defense peptides similarly suppress transcriptional responses to bacterial lipopolysaccharide. J Leukoc Biol (2006) 80(6):1563–74. doi: 10.1189/jlb.0106048 16943385

[47] Bowdish DM Davidson DJ Lau YE Lee K Scott MG Hancock RE. Impact of LL-37 on anti-infective immunity. J Leukoc Biol (2005) 77(4):451–9. doi: 10.1189/jlb.0704380 15569695

[48] Mookherjee N Rehaume LM Hancock RE. Cathelicidins and functional analogues as antisepsis molecules. Expert Opin Ther Targets (2007) 11(8):993–1004. doi: 10.1517/14728222.11.8.993 17665972

[49] Dosler S Karaaslan E. Inhibition and destruction of pseudomonas aeruginosa biofilms by antibiotics and antimicrobial peptides. Peptides (2014) 62:32–7. doi: 10.1016/j.peptides.2014.09.021 25285879

[50] Mookherjee N Anderson MA Haagsman HP Davidson DJ. Antimicrobial host defence peptides: Functions and clinical potential. Nat Rev Drug Discov (2020) 19(5):311–32. doi: 10.1038/s41573-019-0058-8 32107480

[51] Takiguchi T Morizane S Yamamoto T Kajita A Ikeda K Iwatsuki K. Cathelicidin antimicrobial peptide LL-37 augments interferon-β expression and antiviral activity induced by double-stranded RNA in keratinocytes. Br J Dermatol (2014) 171(3):492–8. doi: 10.1111/bjd.12942 24601852

[52] Wei X Zhang L Yang Y Hou Y Xu Y Wang Z. LL-37 transports immunoreactive cGAMP to activate STING signaling and enhance interferon-mediated host antiviral immunity. Cell Rep (2022) 39(9):110880. doi: 10.1016/j.celrep.2022.110880 35649354

[53] Yu J Dai Y Fu Y Wang K Yang Y Li M. Cathelicidin antimicrobial peptides suppress EV71 infection *via* regulating antiviral response and inhibiting viral binding. Antiviral Res (2021) 187:105021. doi: 10.1016/j.antiviral.2021.105021 33508330

[54] Gordon YJ Huang LC Romanowski EG Yates KA Proske RJ McDermott AM. Human cathelicidin (LL-37), a multifunctional peptide, is expressed by ocular surface epithelia and has potent antibacterial and antiviral activity. Curr Eye Res (2005) 30(5):385–94. doi: 10.1080/02713680590934111 PMC1497871 16020269

[55] Wang C Wang S Li D Chen P Han S Zhao G. Human cathelicidin inhibits SARS-CoV-2 infection: Killing two birds with one stone. ACS Infect Dis (2021) 7(6):1545–54. doi: 10.1021/acsinfecdis.1c00096 33849267

[56] Hilchie AL Wuerth K Hancock RE. Immune modulation by multifaceted cationic host defense (antimicrobial) peptides. Nat Chem Biol (2013) 9(12):761–8. doi: 10.1038/nchembio.1393 24231617

[57] Berkestedt I Herwald H Ljunggren L Nelson A Bodelsson M. Elevated plasma levels of antimicrobial polypeptides in patients with severe sepsis. J Innate Immun (2010) 2(5):478–82. doi: 10.1159/000317036 20571257

[58] Neumann A Berends ET Nerlich A Molhoek EM Gallo RL Meerloo T. The antimicrobial peptide LL-37 facilitates the formation of neutrophil extracellular traps. Biochem J (2014) 464(1):3–11. doi: 10.1042/BJ20140778 25181554

[59] Tripathi S Verma A Kim EJ White MR Hartshorn KL. LL-37 modulates human neutrophil responses to influenza a virus. J Leukoc Biol (2014) 96(5):931–8. doi: 10.1189/jlb.4A1113-604RR PMC4197563 25082153

[60] Lande R Botti E Jandus C Dojcinovic D Fanelli G Conrad C. The antimicrobial peptide LL37 is a T-cell autoantigen in psoriasis. Nat Commun (2014) 5:5621. doi: 10.1038/ncomms6621 25470744

[61] Subramanian H Gupta K Guo Q Price R Ali H. Mas-related gene X2 (MrgX2) is a novel G protein-coupled receptor for the antimicrobial peptide LL-37 in human mast cells: Resistance to receptor phosphorylation, desensitization, and internalization. J Biol Chem (2011) 286(52):44739–49. doi: 10.1074/jbc.M111.277152 PMC3247983 22069323

[62] Babolewska E Brzezinska-Blaszczyk E. Human-derived cathelicidin LL-37 directly activates mast cells to proinflammatory mediator synthesis and migratory response. Cell Immunol (2015) 293(2):67–73. doi: 10.1016/j.cellimm.2014.12.006 25577339

[63] Schiemann F Brandt E Gross R Lindner B Mittelstädt J Sommerhoff CP. The cathelicidin LL-37 activates human mast cells and is degraded by mast cell tryptase: Counter-regulation by CXCL4. J Immunol (2009) 183(4):2223–31. doi: 10.4049/jimmunol.0803587 19625657

[64] Agier J Brzezińska-Błaszczyk E Różalska S Wiktorska M Kozłowska E Żelechowska P. Mast cell phenotypic plasticity and their activity under the influence of cathelicidin-related antimicrobial peptide (CRAMP) and IL-33 alarmins. Cell Immunol (2021) 369:104424. doi: 10.1016/j.cellimm.2021.104424 34469845

[65] Agier J Brzezińska-Błaszczyk E Żelechowska P Wiktorska M Pietrzak J Różalska S. Cathelicidin LL-37 affects surface and intracellular toll-like receptor expression in tissue mast cells. J Immunol Res (2018) 2018:7357162. doi: 10.1155/2018/7357162 29670923 PMC5836302

[66] Pena OM Afacan N Pistolic J Chen C Madera L Falsafi R. Synthetic cationic peptide IDR-1018 modulates human macrophage differentiation. PloS One (2013) 8(1):e52449. doi: 10.1371/journal.pone.0052449 23308112 PMC3538731

[67] Chen S Lu Z Wang F Wang Y. Cathelicidin-WA polarizes e. coli K88-induced M1 macrophage to M2-like macrophage in RAW264.7 cells. Int Immunopharmacol (2018) 54:52–9. doi: 10.1016/j.intimp.2017.10.013 29101873

[68] van der Does AM Beekhuizen H Ravensbergen B Vos T Ottenhoff TH van Dissel JT. LL-37 directs macrophage differentiation toward macrophages with a proinflammatory signature. J Immunol (2010) 185(3):1442–9. doi: 10.4049/jimmunol.1000376 20610648

[69] Nijnik A Pistolic J Filewod NC Hancock RE. Signaling pathways mediating chemokine induction in keratinocytes by cathelicidin LL-37 and flagellin. J Innate immun (2012) 4(4):377–86. doi: 10.1159/000335901 PMC6741628 22516952

[70] Kandler K Shaykhiev R Kleemann P Klescz F Lohoff M Vogelmeier C. The anti-microbial peptide LL-37 inhibits the activation of dendritic cells by TLR ligands. Int Immunol (2006) 18(12):1729–36. doi: 10.1093/intimm/dxl107 17041145

[71] Mookherjee N Brown KL Bowdish DM Doria S Falsafi R Hokamp K. Modulation of the TLR-mediated inflammatory response by the endogenous human host defense peptide LL-37. J Immunol (2006) 176(4):2455–64. doi: 10.4049/jimmunol.176.4.2455 16456005

[72] Hu Z Murakami T Suzuki K Tamura H Kuwahara-Arai K Iba T. Antimicrobial cathelicidin peptide LL-37 inhibits the LPS/ATP-induced pyroptosis of macrophages by dual mechanism. PloS One (2014) 9(1):e85765. doi: 10.1371/journal.pone.0085765 24454930 PMC3894207

[73] Larrick JW Hirata M Balint RF Lee J Zhong J Wright SC. Human CAP18: a novel antimicrobial lipopolysaccharide-binding protein. Infect Immun (1995) 63(4):1291–7. doi: 10.1128/iai.63.4.1291-1297.1995 PMC173149 7890387

[74] Ganguly D Chamilos G Lande R Gregorio J Meller S Facchinetti V. Self-RNA-antimicrobial peptide complexes activate human dendritic cells through TLR7 and TLR8. J Exp Med (2009) 206(9):1983–94. doi: 10.1084/jem.20090480 PMC2737167 19703986

[75] Yu K Lai BF Gani J Mikut R Hilpert K Kizhakkedathu JN. Interaction of blood components with cathelicidins and their modified versions. Biomaterials (2015) 69:201–11. doi: 10.1016/j.biomaterials.2015.08.003 26295533

[76] Su W Chen Y Wang C Ding X Rwibasira G Kong Y. Human cathelicidin LL-37 inhibits platelet aggregation and thrombosis *via* Src/PI3K/Akt signaling. Biochem Biophys Res Commun (2016) 473(1):283–9. doi: 10.1016/j.bbrc.2016.03.095 27012197

[77] Johansson J Gudmundsson GH Rottenberg ME Berndt KD Agerberth B. Conformation-dependent antibacterial activity of the naturally occurring human peptide LL-37. J Biol Chem (1998) 273(6):3718–24. doi: 10.1074/jbc.273.6.3718 9452503

[78] Salamah MF Ravishankar D Kodji X Moraes LA Williams HF Vallance TM. The endogenous antimicrobial cathelicidin LL37 induces platelet activation and augments thrombus formation. Blood Adv (2018) 2(21):2973–85. doi: 10.1182/bloodadvances.2018021758 PMC6234361 30413433

[79] Malm J Sorensen O Persson T Frohm-Nilsson M Johansson B Bjartell A. The human cationic antimicrobial protein (hCAP-18) is expressed in the epithelium of human epididymis, is present in seminal plasma at high concentrations, and is attached to spermatozoa. Infect Immun (2000) 68(7):4297–302. doi: 10.1128/IAI.68.7.4297-4302.2000 PMC101750 10858248

[80] Frigimelica E Bartolini E Galli G Grandi G Grifantini R. Identification of 2 hypothetical genes involved in neisseria meningitidis cathelicidin resistance. J Infect Dis (2008) 197(8):1124–32. doi: 10.1086/533456 18462162

[81] Bergman P Johansson L Wan H Jones A Gallo RL Gudmundsson GH. Induction of the antimicrobial peptide CRAMP in the blood-brain barrier and meninges after meningococcal infection. Infect Immun (2006) 74(12):6982–91. doi: 10.1128/IAI.01043-06 PMC1698100 17030578

[82] Schaller-Bals S Schulze A Bals R. Increased levels of antimicrobial peptides in tracheal aspirates of newborn infants during infection. Am J Respir Crit Care Med (2002) 165(7):992–5. doi: 10.1164/ajrccm.165.7.200110-020 11934727

[83] Wan M Godson C Guiry PJ Agerberth B Haeggström JZ. Leukotriene B4/antimicrobial peptide LL-37 proinflammatory circuits are mediated by BLT1 and FPR2/ALX and are counterregulated by lipoxin A4 and resolvin E1. FASEB J (2011) 25(5):1697–705. doi: 10.1096/fj.10-175687 21307335

[84] Iaccio A Cattaneo F Mauro M Ammendola R. FPRL1-mediated induction of superoxide in LL-37-stimulated IMR90 human fibroblast. Arch Biochem Biophys (2009) 481(1):94–100. doi: 10.1016/j.abb.2008.10.026 18996352

[85] Czapiga M Gao JL Kirk A Lekstrom-Himes J. Human platelets exhibit chemotaxis using functional n-formyl peptide receptors. Exp hematol (2005) 33(1):73–84. doi: 10.1016/j.exphem.2004.09.010 15661400

[86] Rowley JW Oler AJ Tolley ND Hunter BN Low EN Nix DA. Genome-wide RNA-seq analysis of human and mouse platelet transcriptomes. Blood (2011) 118(14):e101–11. doi: 10.1182/blood-2011-03-339705 PMC3193274 21596849

[87] Salamah MF Vallance TM Kodji X Ravishankar D Williams HF Brain SD. The antimicrobial cathelicidin CRAMP augments platelet activation during psoriasis in mice. Biomolecules (2020) 10(9). doi: 10.3390/biom10091267 PMC7565973 32887440

[88] Sánchez-Peña FJ Romero-Tlalolini M Torres-Aguilar H Cruz-Hernández DS Baltiérrez-Hoyos R Sánchez-Aparicio SR. LL-37 triggers antimicrobial activity in human platelets. Int J Mol Sci (2023) 24(3). doi: 10.3390/ijms24032816 PMC9917488 36769137

[89] Aloul KM Nielsen JE Defensor EB Lin JS Fortkort JA Shamloo M. Upregulating human cathelicidin antimicrobial peptide LL-37 expression may prevent severe COVID-19 inflammatory responses and reduce microthrombosis. Front Immunol (2022) 13:880961. doi: 10.3389/fimmu.2022.880961 35634307 PMC9134243

[90] Creel-Bulos C Hockstein M Amin N Melhem S Truong A Sharifpour M. Acute cor pulmonale in critically ill patients with covid-19. N Engl J Med (2020) 382(21):e70. doi: 10.1056/NEJMc2010459 32374956 PMC7281714

[91] Duan Z Zhang J Chen X Liu M Zhao H Jin L. Role of LL-37 in thrombotic complications in patients with COVID-19. Cell Mol Life Sci (2022) 79(6):309. doi: 10.1007/s00018-022-04309-y 35596804 PMC9123294

[92] Andersson E Rydengård V Sonesson A Mörgelin M Björck L Schmidtchen A. Antimicrobial activities of heparin-binding peptides. Eur J Biochem (2004) 271(6):1219–26. doi: 10.1111/j.1432-1033.2004.04035.x 15009200

[93] Swystun LL Liaw PC. The role of leukocytes in thrombosis. Blood (2016) 128(6):753–62. doi: 10.1182/blood-2016-05-718114 27354721

[94] Neumann A Vollger L Berends ET Molhoek EM Stapels DA Midon M. Novel role of the antimicrobial peptide LL-37 in the protection of neutrophil extracellular traps against degradation by bacterial nucleases. J Innate Immun (2014) 6(6):860–8. doi: 10.1159/000363699 PMC4201878 25012862

[95] Kolaczkowska E Kubes P. Neutrophil recruitment and function in health and inflammation. Nat Rev Immunol (2013) 13(3):159–75. doi: 10.1038/nri3399 23435331

[96] Massberg S Grahl L von Bruehl ML Manukyan D Pfeiler S Goosmann C. Reciprocal coupling of coagulation and innate immunity *via* neutrophil serine proteases. Nat Med (2010) 16(8):887–96. doi: 10.1038/nm.2184 20676107

[97] Sørensen OE Follin P Johnsen AH Calafat J Tjabringa GS Hiemstra PS. Human cathelicidin, hCAP-18, is processed to the antimicrobial peptide LL-37 by extracellular cleavage with proteinase 3. Blood (2001) 97(12):3951–9. doi: 10.1182/blood.V97.12.3951 11389039

[98] Lande R Ganguly D Facchinetti V Frasca L Conrad C Gregorio J. Neutrophils activate plasmacytoid dendritic cells by releasing self-DNA-peptide complexes in systemic lupus erythematosus. Sci Trans Med (2011) 3(73):73ra19. doi: 10.1126/scitranslmed.3001180 PMC3399524 21389263

[99] Kolaczkowska E Jenne CN Surewaard BG Thanabalasuriar A Lee WY Sanz MJ. Molecular mechanisms of NET formation and degradation revealed by intravital imaging in the liver vasculature. Nat Commun (2015) 6:6673. doi: 10.1038/ncomms7673 25809117 PMC4389265

[100] Lande R Gregorio J Facchinetti V Chatterjee B Wang YH Homey B. Plasmacytoid dendritic cells sense self-DNA coupled with antimicrobial peptide. Nature (2007) 449(7162):564–9. doi: 10.1038/nature06116 17873860

[101] Luo D Szaba FM Kummer LW Plow EF Mackman N Gailani D. Protective roles for fibrin, tissue factor, plasminogen activator inhibitor-1, and thrombin activatable fibrinolysis inhibitor, but not factor XI, during defense against the gram-negative bacterium yersinia enterocolitica. J Immunol (2011) 187(4):1866–76. doi: 10.4049/jimmunol.1101094 PMC3150340 21724997

[102] Stark K Philippi V Stockhausen S Busse J Antonelli A Miller M. Disulfide HMGB1 derived from platelets coordinates venous thrombosis in mice. Blood (2016) 128(20):2435–49. doi: 10.1182/blood-2016-04-710632 PMC5147023 27574188

[103] Wantha S Alard J-E Megens RTA Does A Döring Y Drechsler M. Neutrophil-derived cathelicidin promotes adhesion of classical monocytes. Circ Res (2013) 112(5):792–801. doi: 10.1161/CIRCRESAHA.112.300666 23283724 PMC3702173

[104] Minns D Smith KJ Alessandrini V Hardisty G Melrose L Jackson-Jones L. The neutrophil antimicrobial peptide cathelicidin promotes Th17 differentiation. Nat Commun (2021) 12(1):1285. doi: 10.1038/s41467-021-21533-5 33627652 PMC7904761

[105] Taleb S Tedgui A Mallat Z. IL-17 and Th17 cells in atherosclerosis: subtle and contextual roles. Arterioscler Thromb Vasc Biol (2015) 35(2):258–64. doi: 10.1161/ATVBAHA.114.303567 25234818

[106] Cremoni M Brglez V Perez S Decoupigny F Zorzi K Andreani M. Th17-immune response in patients with membranous nephropathy is associated with thrombosis and relapses. Front Immunol (2020) 11:574997. doi: 10.3389/fimmu.2020.574997 33324398 PMC7725714

[107] Chernomordik F Cercek B Lio WM Mihailovic PM Yano J Herscovici R. The role of T cells reactive to the cathelicidin antimicrobial peptide LL-37 in acute coronary syndrome and plaque calcification. Front Immunol (2020) 11:575577. doi: 10.3389/fimmu.2020.575577 33123157 PMC7573569

[108] Furchgott RF Vanhoutte PM. Endothelium-derived relaxing and contracting factors. FASEB J (1989) 3(9):2007–18. doi: 10.1096/fasebj.3.9.2545495 2545495

[109] Furchgott RF Zawadzki JV. The obligatory role of endothelial cells in the relaxation of arterial smooth muscle by acetylcholine. Nature (1980) 288(5789):373–6. doi: 10.1038/288373a0 6253831

[110] Moncada S Palmer RM Higgs EA. Nitric oxide: physiology, pathophysiology, and pharmacology. Pharmacol Rev (1991) 43(2):109–42. 1852778

[111] Radomski MW Palmer RM Moncada S. Endogenous nitric oxide inhibits human platelet adhesion to vascular endothelium. Lancet (1987) 2(8567):1057–8. doi: 10.1016/S0140-6736(87)91481-4 2889967

[112] Downing LJ Wakefield TW Strieter RM Prince MR Londy FJ Fowlkes JB. Anti-p-selectin antibody decreases inflammation and thrombus formation in venous thrombosis. J Vasc Surg (1997) 25(5):816–27. doi: 10.1016/S0741-5214(97)70211-8 9152309

[113] Cai H Harrison DG. Endothelial dysfunction in cardiovascular diseases: the role of oxidant stress. Circ Res (2000) 87(10):840–4. doi: 10.1161/01.RES.87.10.840 11073878

[114] Stenvinkel P. Endothelial dysfunction and inflammation-is there a link? Nephrol Dial Transplant (2001) 16(10):1968–71. doi: 10.1093/ndt/16.10.1968 11572879

[115] Suzuki K Ohkuma M Nagaoka I. Bacterial lipopolysaccharide and antimicrobial LL-37 enhance ICAM-1 expression and NF-κB p65 phosphorylation in senescent endothelial cells. Int J Mol Med (2019) 44(4):1187–96. doi: 10.3892/ijmm.2019.4294 PMC6713406 31364735

[116] Edfeldt K Agerberth B Rottenberg ME Gudmundsson GH Wang XB Mandal K. Involvement of the antimicrobial peptide LL-37 in human atherosclerosis. Arterioscler Thromb Vasc Biol (2006) 26(7):1551–7. doi: 10.1161/01.ATV.0000223901.08459.57 16645154

[117] Ju Y Hua J Sakamoto K Ogawa H Nagaoka I. Glucosamine, a naturally occurring amino monosaccharide modulates LL-37-induced endothelial cell activation. Int J Mol Med (2008) 22(5):657–62. doi: 10.3892/ijmm_00000069 18949387

[118] Suzuki K Ohkuma M Someya A Mita T Nagaoka I. Human cathelicidin peptide LL-37 induces cell death in autophagy-dysfunctional endothelial cells. J Immunol (2022) 208(9):2163–72. doi: 10.4049/jimmunol.2100050 PMC9047070 35387840

[119] Merkle M Pircher J Mannell H Krotz F Blum P Czermak T. LL37 inhibits the inflammatory endothelial response induced by viral or endogenous DNA. J Autoimmun (2015) 65:19–29. doi: 10.1016/j.jaut.2015.07.015 26297208

[120] Koczulla R von Degenfeld G Kupatt C Krotz F Zahler S Gloe T. An angiogenic role for the human peptide antibiotic LL-37/hCAP-18. J Clin Invest (2003) 111(11):1665–72. doi: 10.1172/JCI17545 PMC156109 12782669

[121] Salvado MD Di Gennaro A Lindbom L Agerberth B Haeggström JZ. Cathelicidin LL-37 induces angiogenesis *via* PGE2-EP3 signaling in endothelial cells, *in vivo* inhibition by aspirin. Arterioscler Thromb Vasc Biol (2013) 33(8):1965–72. doi: 10.1161/ATVBAHA.113.301851 23766266

[122] Szulcek R Bollensdorff C Hordijk P Gabriel M. The covalently immobilized antimicrobial peptide LL37 acts as a VEGF mimic and stimulates endothelial cell proliferation. Biochem Biophys Res Commun (2018) 496(3):887–90. doi: 10.1016/j.bbrc.2018.01.130 29366792

[123] Coffelt SB Marini FC Watson K Zwezdaryk KJ Dembinski JL LaMarca HL. The pro-inflammatory peptide LL-37 promotes ovarian tumor progression through recruitment of multipotent mesenchymal stromal cells. Proc Natl Acad Sci USA (2009) 106(10):3806–11. doi: 10.1073/pnas.0900244106 PMC2656161 19234121

[124] Ramos R Silva JP Rodrigues AC Costa R Guardão L Schmitt F. Wound healing activity of the human antimicrobial peptide LL37. Peptides (2011) 32(7):1469–76. doi: 10.1016/j.peptides.2011.06.005 21693141

[125] Soehnlein O Wantha S Simsekyilmaz S Doring Y Megens RT Mause SF. Neutrophil-derived cathelicidin protects from neointimal hyperplasia. Sci Trans Med (2011) 3(103):103ra98. doi: 10.1126/scitranslmed.3002531 PMC3246402 21974936

[126] Werner N Junk S Laufs U Link A Walenta K Bohm M. Intravenous transfusion of endothelial progenitor cells reduces neointima formation after vascular injury. Circ Res (2003) 93(2):e17–24. doi: 10.1161/01.RES.0000083812.30141.74 12829619

[127] Berkestedt I Nelson A Bodelsson M. Endogenous antimicrobial peptide LL-37 induces human vasodilatation. Br J Anaesth (2008) 100(6):803–9. doi: 10.1093/bja/aen074 18397922

[128] Wu H Zhang G Minton JE Ross CR Blecha F. Regulation of cathelicidin gene expression: induction by lipopolysaccharide, interleukin-6, retinoic acid, and salmonella enterica serovar typhimurium infection. Infect Immun (2000) 68(10):5552–8. doi: 10.1128/IAI.68.10.5552-5558.2000 PMC101505 10992453

[129] Kahlenberg JM Kaplan MJ. Little peptide, big effects: The role of LL-37 in inflammation and autoimmune disease. J Immunol (2013) 191(10):4895–901. doi: 10.4049/jimmunol.1302005 PMC3836506 24185823

[130] Tjabringa GS Rabe KF Hiemstra PS. The human cathelicidin LL-37: A multifunctional peptide involved in infection and inflammation in the lung. Pulm Pharmacol Ther (2005) 18(5):321–7. doi: 10.1016/j.pupt.2005.01.001 15939310

[131] Van Harten RM Van Woudenbergh E Van Dijk A Haagsman HP. Cathelicidins: Immunomodulatory antimicrobials. Vaccines (2018) 6(3):63. doi: 10.3390/vaccines6030063 30223448 PMC6161271

[132] Kai-Larsen Y Agerberth B. The role of the multifunctional peptide LL-37 in host defense. Front Bioscience-Landmark (2008) 13(10):3760–7. doi: 10.2741/2964 18508470

[133] Park J Kim M Kang SG Jannasch AH Cooper B Patterson J. Short-chain fatty acids induce both effector and regulatory T cells by suppression of histone deacetylases and regulation of the mTOR–S6K pathway. Mucosal Immunol (2015) 8(1):80–93. doi: 10.1038/mi.2014.44 24917457 PMC4263689

[134] Maugeri N Campana L Gavina M Covino C De Metrio M Panciroli C. Activated platelets present high mobility group box 1 to neutrophils, inducing autophagy and promoting the extrusion of neutrophil extracellular traps. J Thromb Haemost (2014) 12(12):2074–88. doi: 10.1111/jth.12710 25163512

[135] Etulain J Martinod K Wong SL Cifuni SM Schattner M Wagner DD. P-selectin promotes neutrophil extracellular trap formation in mice. Blood (2015) 126(2):242–6. doi: 10.1182/blood-2015-01-624023 PMC4497964 25979951

[136] Duerschmied D Suidan GL Demers M Herr N Carbo C Brill A. Platelet serotonin promotes the recruitment of neutrophils to sites of acute inflammation in mice. Blood (2013) 121(6):1008–15. doi: 10.1182/blood-2012-06-437392 PMC3567335 23243271

[137] Lacy P. Mechanisms of degranulation in neutrophils. Allergy Asthma Clin Immunol (2006) 2(3):98. doi: 10.1186/1710-1492-2-3-98 20525154 PMC2876182

[138] Villanueva E Yalavarthi S Berthier CC Hodgin JB Khandpur R Lin AM. Netting neutrophils induce endothelial damage, infiltrate tissues, and expose immunostimulatory molecules in systemic lupus erythematosus. J Immunol (2011) 187(1):538–52. doi: 10.4049/jimmunol.1100450 PMC3119769 21613614

[139] Ciornei CD Tapper H Bjartell A Sternby NH Bodelsson M. Human antimicrobial peptide LL-37 is present in atherosclerotic plaques and induces death of vascular smooth muscle cells: a laboratory study. BMC Cardiovasc Disord (2006) 6:49. doi: 10.1186/1471-2261-6-49 17181861 PMC1764755

[140] Zhang Z Meng P Han Y Shen C Li B Hakim MA. Mitochondrial DNA-LL-37 complex promotes atherosclerosis by escaping from autophagic recognition. Immunity (2015) 43(6):1137–47. doi: 10.1016/j.immuni.2015.10.018 26680206

[141] Zhao H Yan H Yamashita S Li W Liu C Chen Y. Acute ST-segment elevation myocardial infarction is associated with decreased human antimicrobial peptide LL-37 and increased human neutrophil peptide-1 to 3 in plasma. J Atheroscl Thromb (2012) 19(4):357–68. doi: 10.5551/jat.10108 22186100

[142] Bei Y Pan LL Zhou Q Zhao C Xie Y Wu C. Cathelicidin-related antimicrobial peptide protects against myocardial ischemia/reperfusion injury. BMC Med (2019) 17(1):42. doi: 10.1186/s12916-019-1268-y 30782145 PMC6381635

[143] Wu Y Zhang Y Zhang J Zhai T Hu J Luo H. Cathelicidin aggravates myocardial ischemia/reperfusion injury *via* activating TLR4 signaling and P2X(7)R/NLRP3 inflammasome. J Mol Cell Cardiol (2020) 139:75–86. doi: 10.1016/j.yjmcc.2019.12.011 31982429

[144] Zhao H Sheng Z Tan Y Chen R Zhou J Li J. High human antimicrobial peptide LL-37 level predicts lower major adverse cardiovascular events after an acute ST-segment elevation myocardial infarction. J Atheroscl Thromb (2022) 29(10):1499–510. doi: 10.5551/jat.63221 PMC9529390 34853213

[145] Wu W Kim CH Liu R Kucia M Marlicz W Greco N. The bone marrow-expressed antimicrobial cationic peptide LL-37 enhances the responsiveness of hematopoietic stem progenitor cells to an SDF-1 gradient and accelerates their engraftment after transplantation. Leukemia (2012) 26(4):736–45. doi: 10.1038/leu.2011.252 PMC3244577 21931324

[146] Karapetyan AV Klyachkin YM Selim S Sunkara M Ziada KM Cohen DA. Bioactive lipids and cationic antimicrobial peptides as new potential regulators for trafficking of bone marrow-derived stem cells in patients with acute myocardial infarction. Stem Cells Dev (2013) 22(11):1645–56. doi: 10.1089/scd.2012.0488 PMC3657281 23282236

[147] Klyachkin YM Idris A Rodell CB Tripathi H Ye S Nagareddy P. Cathelicidin related antimicrobial peptide (CRAMP) enhances bone marrow cell retention and attenuates cardiac dysfunction in a mouse model of myocardial infarction. Stem Cell Rev Rep (2018) 14(5):702–14. doi: 10.1007/s12015-018-9833-x PMC6119631 29948752

[148] Kumagai Y Murakami T Kuwahara A Iba T Reich J Nagaoka I. Antimicrobial peptide LL-37 ameliorates a murine sepsis model *via* the induction of microvesicle release from neutrophils. Innate Immun (2020) 26(7):565–79. doi: 10.1177/1753425920936754 PMC7556193 32600088

[149] Hosoda H Nakamura K Hu Z Tamura H Reich J Kuwahara-Arai K. Antimicrobial cathelicidin peptide LL−37 induces NET formation and suppresses the inflammatory response in a mouse septic model. Mol Med Rep (2017) 16(4):5618–26. doi: 10.3892/mmr.2017.7267 28849130

[150] Kahlenberg JM Carmona-Rivera C Smith CK Kaplan MJ. Neutrophil extracellular trap-associated protein activation of the NLRP3 inflammasome is enhanced in lupus macrophages. J Immunol (2013) 190(3):1217–26. doi: 10.4049/jimmunol.1202388 PMC3552129 23267025

[151] Keutmann M Hermes G Meinberger D Roth A Stemler J Cornely OA. The ratio of serum LL-37 levels to blood leucocyte count correlates with COVID-19 severity. Sci Rep (2022) 12(1):9447. doi: 10.1038/s41598-022-13260-8 35676519 PMC9175165

[152] Drechsler M Megens RT van Zandvoort M Weber C Soehnlein O. Hyperlipidemia-triggered neutrophilia promotes early atherosclerosis. Circulation (2010) 122(18):1837–45. doi: 10.1161/CIRCULATIONAHA.110.961714 20956207

[153] Mangold A Alias S Scherz T Hofbauer T Jakowitsch J Panzenbock A. Coronary neutrophil extracellular trap burden and deoxyribonuclease activity in ST-elevation acute coronary syndrome are predictors of ST-segment resolution and infarct size. Circ Res (2015) 116(7):1182–92. doi: 10.1161/CIRCRESAHA.116.304944 25547404

[154] Riegger J Byrne RA Joner M Chandraratne S Gershlick AH Ten Berg JM. Histopathological evaluation of thrombus in patients presenting with stent thrombosis. a multicenter European study: a report of the prevention of late stent thrombosis by an interdisciplinary global European effort consortium. Eur Heart J (2016) 37(19):1538–49. doi: 10.1093/eurheartj/ehv419 PMC4872283 26761950

[155] Silvestre-Roig C Braster Q Ortega-Gomez A Soehnlein O. Neutrophils as regulators of cardiovascular inflammation. Nat Rev Cardiol (2020) 17(6):327–40. doi: 10.1038/s41569-019-0326-7 31996800

[156] Silvestre-Roig C Braster Q Wichapong K Lee EY Teulon JM Berrebeh N. Externalized histone H4 orchestrates chronic inflammation by inducing lytic cell death. Nature (2019) 569(7755):236–40. doi: 10.1038/s41586-019-1167-6 PMC6716525 31043745

[157] Petzold T Zhang Z Ballesteros I Saleh I Polzin A Thienel M. Neutrophil “plucking” on megakaryocytes drives platelet production and boosts cardiovascular disease. Immunity (2022) 55(12):2285–99.e7. doi: 10.1016/j.immuni.2022.10.001 36272416 PMC9767676

[158] Fuchs TA Brill A Duerschmied D Schatzberg D Monestier M Myers DD Jr.. Extracellular DNA traps promote thrombosis. Proc Natl Acad Sci USA (2010) 107(36):15880–5. doi: 10.1073/pnas.1005743107 PMC2936604 20798043

[159] Martinod K Deppermann C. Immunothrombosis and thromboinflammation in host defense and disease. Platelets (2021) 32(3):314–24. doi: 10.1080/09537104.2020.1817360 32896192

[160] Esmon CT Esmon NL. The link between vascular features and thrombosis. Annu Rev Physiol (2011) 73:503–14. doi: 10.1146/annurev-physiol-012110-142300 20887194

[161] Esmon CT Xu J Lupu F. Innate immunity and coagulation. J Thromb Haemost (2011) 9 Suppl 1(Suppl 1):182–8. doi: 10.1111/j.1538-7836.2011.04323.x PMC3151110 21781254

[162] Meyers S Crescente M Verhamme P Martinod K. Staphylococcus aureus and neutrophil extracellular traps: The master manipulator meets its match in immunothrombosis. Arterioscler Thromb Vasc Biol (2022) 42(3):261–76. doi: 10.1161/ATVBAHA.121.316930 PMC8860219 35109674

[163] Frantzeskaki F Armaganidis A Orfanos SE. Immunothrombosis in acute respiratory distress syndrome: Cross talks between inflammation and coagulation. Respiration (2017) 93(3):212–25. doi: 10.1159/000453002 27997925

[164] Bray MA Sartain SE Gollamudi J Rumbaut RE. Microvascular thrombosis: experimental and clinical implications. Transl Res (2020) 225:105–30. doi: 10.1016/j.trsl.2020.05.006 PMC7245314 32454092

[165] Kahlenberg JM Thacker SG Berthier CC Cohen CD Kretzler M Kaplan MJ. Inflammasome activation of IL-18 results in endothelial progenitor cell dysfunction in systemic lupus erythematosus. J Immunol (2011) 187(11):6143–56. doi: 10.4049/jimmunol.1101284 PMC3221936 22058412

[166] Visser MJE Tarr G Pretorius E. Thrombosis in psoriasis: Cutaneous cytokine production as a potential driving force of haemostatic dysregulation and subsequent cardiovascular risk. Front Immunol (2021) 12:688861. doi: 10.3389/fimmu.2021.688861 34335591 PMC8324086

[167] Nicolai L Leunig A Brambs S Kaiser R Weinberger T Weigand M. Immunothrombotic dysregulation in COVID-19 pneumonia is associated with respiratory failure and coagulopathy. Circulation (2020) 142(12):1176–89. doi: 10.1161/CIRCULATIONAHA.120.048488 PMC7497892 32755393

[168] Middleton EA He XY Denorme F Campbell RA Ng D Salvatore SP. Neutrophil extracellular traps contribute to immunothrombosis in COVID-19 acute respiratory distress syndrome. Blood (2020) 136(10):1169–79. doi: 10.1182/blood.2020007008 PMC7472714 32597954

[169] Skendros P Mitsios A Chrysanthopoulou A Mastellos DC Metallidis S Rafailidis P. Complement and tissue factor-enriched neutrophil extracellular traps are key drivers in COVID-19 immunothrombosis. J Clin Invest (2020) 130(11):6151–7. doi: 10.1172/JCI141374 PMC7598040 32759504

[170] Lokhande KB Banerjee T Swamy KV Ghosh P Deshpande M. An in silico scientific basis for LL-37 as a therapeutic for covid-19. Proteins (2022) 90(5):1029–43. doi: 10.1002/prot.26198 PMC8441666 34333809

[171] Nireeksha N Gollapalli P Varma SR Hegde MN Kumari NS. Utilizing the potential of antimicrobial peptide LL-37 for combating SARS-COV- 2 viral load in saliva: an in silico analysis. Eur J Dent (2022) 16(3):478–87. doi: 10.1055/s-0041-1739444 PMC9507610 34937110

[172] Saiman L Tabibi S Starner TD San Gabriel P Winokur PL Jia HP. Cathelicidin peptides inhibit multiply antibiotic-resistant pathogens from patients with cystic fibrosis. Antimicrob Agents Chemother (2001) 45(10):2838–44. doi: 10.1128/AAC.45.10.2838-2844.2001 PMC90740 11557478

[173] Bals R Weiner DJ Meegalla RL Wilson JM. Transfer of a cathelicidin peptide antibiotic gene restores bacterial killing in a cystic fibrosis xenograft model. J Clin Invest (1999) 103(8):1113–7. doi: 10.1172/JCI6570 PMC408283 10207162

[174] Duplantier AJ van Hoek ML. The human cathelicidin antimicrobial peptide LL-37 as a potential treatment for polymicrobial infected wounds. Front Immunol (2013) 4:143. doi: 10.3389/fimmu.2013.00143 23840194 PMC3699762

[175] Wnorowska U Fiedoruk K Piktel E Prasad SV Sulik M Janion M. Nanoantibiotics containing membrane-active human cathelicidin LL-37 or synthetic ceragenins attached to the surface of magnetic nanoparticles as novel and innovative therapeutic tools: current status and potential future applications. J Nanobiotechnol (2020) 18(1):3. doi: 10.1186/s12951-019-0566-z PMC6939332 31898542

[176] Niemirowicz K Durnaś B Tokajuk G Piktel E Michalak G Gu X. Formulation and candidacidal activity of magnetic nanoparticles coated with cathelicidin LL-37 and ceragenin CSA-13. Sci Rep (2017) 7(1):4610. doi: 10.1038/s41598-017-04653-1 28676673 PMC5496903

[177] Lee CJ Buznyk O Kuffova L Rajendran V Forrester JV Phopase J. Cathelicidin LL-37 and HSV-1 corneal infection: Peptide versus gene therapy. Transl Vis Sci Technol (2014) 3(3):4. doi: 10.1167/tvst.3.3.4 PMC4043105 24932432

[178] Grönberg A Mahlapuu M Ståhle M Whately-Smith C Rollman O. Treatment with LL-37 is safe and effective in enhancing healing of hard-to-heal venous leg ulcers: A randomized, placebo-controlled clinical trial. Wound Repair Regen (2014) 22(5):613–21. doi: 10.1111/wrr.12211 25041740

[179] Finger S Knorr M Molitor M Schüler R Garlapati V Waisman A. A sequential interferon gamma directed chemotactic cellular immune response determines survival and cardiac function post-myocardial infarction. Cardiovasc Res (2019) 115(13):1907–17. doi: 10.1093/cvr/cvz092 30949687

[180] Oostingh GJ Pozgajova M Ludwig RJ Krahn T Boehncke WH Nieswandt B. Diminished thrombus formation and alleviation of myocardial infarction and reperfusion injury through antibody- or small-molecule-mediated inhibition of selectin-dependent platelet functions. Haematologica (2007) 92(4):502–12. doi: 10.3324/haematol.10741 17488661

